# Notable paradoxical phenomena in associations between cardiovascular health score, subclinical and clinical cardiovascular disease in the community: The Framingham Heart Study

**DOI:** 10.1371/journal.pone.0267267

**Published:** 2022-05-05

**Authors:** Maximillian T. Bourdillon, Bamba Gaye, Rebecca J. Song, Ramachandran S. Vasan, Vanessa Xanthakis

**Affiliations:** 1 University of Texas Health Science Center at Houston, Houston, TX, United States of America; 2 INSERM, U970, Paris Cardiovascular Research Center, University Paris Descartes, Sorbonne Paris Cité, Paris, France; 3 Department of Epidemiology, Boston University School of Public Health, Boston, MA, United States of America; 4 Department of Medicine, Section of Preventive Medicine and Epidemiology, Boston University School of Medicine, Boston, MA, United States of America; 5 Section of Cardiology, Boston University School of Medicine, Boston, MA, United States of America; 6 Center for Computing and Data Sciences, Boston University, Boston, MA, United States of America; 7 Boston University’s and National Heart, Lung, and Blood Institute’s Framingham Heart Study, Framingham, MA, United States of America; 8 Department of Biostatistics, Boston University School of Public Health, Boston, MA, United States of America; Albert Einstein College of Medicine, UNITED STATES

## Abstract

**Importance:**

Cardiovascular Health (CVH) scores are inversely associated with prevalent subclinical (SubDz) and incident cardiovascular disease (CVD). However, the majority of people who develop CVD have intermediate or ideal CVH scores, while many with poor CVH profiles escape CVD development.

**Objective:**

To describe the prevalence of paradoxical relations among CVH, SubDz, and CVD.

**Design:**

Cohort study, Framingham Study data collected prospectively (1995–2016).

**Setting:**

Population-based.

**Participants:**

7,627 participants (mean age 49 years, 53% women) attending Offspring examinations 6/7 and Third Generation examinations 1/2.

**Exposures:**

CVH score (range 0–14) constructed from poor, intermediate, or ideal status for each metric (smoking, diet, physical activity, blood pressure, body mass index, fasting glucose, total cholesterol); and prevalent SubDz (≥1 of: increased carotid intimal media thickness, CIMT; left ventricular hypertrophy, LVH; microalbuminuria, MA; elevated ankle brachial index, ABI; coronary artery calcium score ≥100,CAC).

**Main outcome(s) and measure(s):**

Ideal CVH (scores 12–14), intermediate CVH (scores 8–11), and poor CVH (0–7). We described three distinct paradoxical phenomena, involving combinations of CVH, SubDz, and CVD, and generated CVD incidence rates and predicted CVD probabilities for all combinations.

**Results:**

We observed 842 CVD events (median follow-up 13.7 years); 1,663 participants had SubDz. Most individuals with poor CVH (78%) or SubDz (57% for CIMT to 77% for LVH) did not develop CVD on follow-up. Among participants with incident CVD, the majority had intermediate or ideal CVH (68%) or absent SubDz (46% for CAC to 96% for ABI) at baseline. We observed similar paradoxical results in relations between CVH and prevalent SubDz. Poor CVH and prevalent SubDz were each associated with higher CVD incidence rates compared to intermediate or ideal CVH and absent SubDz, respectively. The predicted CVD probability was nearly three-times greater among participants with poor (22%) versus intermediate or ideal CVH (8%). Mean CVD predicted probabilities were nearly three (26% vs. 10% for MA) to six-times (29% vs. 5% for CAC) greater among participants with SubDz versus without SubDz. Findings were consistent within age and sex strata.

**Conclusions and relevance:**

Although poor CVH and SubDz presence are associated with CVD incidence, paradoxical phenomena involving CVH, SubDz, and CVD are frequently prevalent in the community. Further studies to elucidate biological mechanisms underlying these phenomena are warranted.

## Introduction

The American Heart Association (AHA) formulated the Life’s Simple 7 Score to measure cardiovascular health (CVH), comprising modifiable health behaviors (smoking, physical activity, diet) and risk factors (body mass index [BMI], blood pressure [BP], serum cholesterol, and fasting glucose) [1]. CVH scores have been inversely associated with odds of subclinical cardiovascular disease (SubDz) and the risk of multiple disease outcomes, including cardiovascular disease (CVD), stroke, neurocognitive impairment, and death [2–11]. Moreover, increased time spent in intermediate or ideal CVH during midlife has been related to a lower risk of cardio-metabolic morbidity and mortality in later life [12]. Despite national efforts to improve CVH, achievement of ideal status on all seven CVH metrics remains low nationally, and may even be declining [13–17].

Although inverse associations of CVH scores with risk of subclinical and clinical CVD have been well reported, data regarding the presence of subclinical or the development of clinical CVD among people with intermediate or ideal CVH are lacking. Furthermore, it is not known whether poor CVH is always accompanied by the presence of subclinical or followed by the development of clinical CVD. Additionally, although the presence of SubDz is associated with a higher risk of CVD [2, 18–26], it is unclear what proportions of people with SubDz will go on to develop CVD. Investigators from the Northern Manhattan Study reported the “Hispanic Paradox”, whereby participants with less favorable risk factor profiles had a lower risk of CVD-related death compared to those with better risk factor profiles [27], but additional and more comprehensive data regarding associations involving CVH, SubDz, and CVD in the community are lacking.

We aimed to describe the presence of potential *paradoxical* relations among CVH, SubDz, and incident CVD using the community-based sample of middle-aged Framingham Heart Study (FHS) participants. More specifically, we focused on three paradoxes: a) *CVH-CVD paradox*, whereby participants with poor CVH do not develop CVD and those with intermediate or ideal CVH develop it; b) *CVH-SubDz paradox*, whereby individuals with poor CVH do not have SubDz and those with intermediate or ideal CVH have SubDz present; and c) *SubDz-CVD paradox*, whereby people with SubDz do not develop CVD and those with absent SubDz develop CVD.

## Methods

### Study sample

The design and selection criteria for the FHS Offspring Study (FOS) and Third Generation Cohorts (Gen3) have been described previously [28, 29]. Our investigation included six distinct study samples of FOS participants who attended examination cycles 6 (1995–1998) or 7 (1998–2001) and Gen3 participants who attended examination cycles 1 (2002–2005) or 2 (2008–2011). Among 3,532 FOS participants who attended examination cycle 6, we excluded 1,084 participants with BMI <18.5 (n = 9), serum creatinine ≥2 mg/dL (n = 13), or missing CVH metrics (n = 1,062), resulting in a sample of 2,448 FOS participants with CVH scores. We further excluded 413 people with prevalent CVD at examination cycle 6, yielding 2,035 participants with available CVH scores to describe the *CVH-CVD paradox* (**Sample 1a**; S1 Fig). Among 3,667 FOS participants with ≥1 SubDz component measured at examination cycle 6 or 7, 2,431 participants also had a CVH score (**Sample 2a**; S1 Fig), which was used to describe the *CVH-SubDz parado*x. To describe the *SubDz-CVD paradox*, we included 3,667 FOS participants with ≥1 SubDz component measurement from examination cycle 6 or 7 and excluded 397 or 439 participants with prevalent CVD at examination cycle 6 or 7, respectively, resulting in a sample of 3,270 total participants (**Sample 3a**; S1 Fig). S1 Fig provides the sub-sample sizes for the five SubDz components within **Sample 3a**.

Additionally, among 4,095 Gen3 participants who attended examination cycle 1, we excluded 749 participants for the following reasons: BMI <18.5 kg/m^2^ (n = 51), serum creatinine ≥2 mg/dL (n = 1), age <25 years (n = 213), or missing CVH metrics (n = 484). These exclusions resulted in a sample of 3,346 Gen3 participants with a CVH score. We further excluded 66 participants with prevalent CVD at examination cycle 1 resulting in 3,280 participants to describe the *CVH-CVD paradox* (**Sample 1b**; S2 Fig). Among 4,093 Gen3 participants with ≥1 SubDz component measured at examination 1 or 2, 3,346 participants also had a CVH score, comprising (**Sample 2b**; S2 Fig), to describe the *CVH-SubDz paradox*. Among the 4,093 Gen3 participants who had ≥1 SubDz component measured, we excluded 66 or 87 participants with prevalent CVD at examination cycle 1 or 2, respectively, which yielded 4,027 total participants (**Sample 3b**; S2 Fig) to describe the *SubDz-CVD paradox*. S2 Fig provides the sub-sample sizes for the four SubDz components within **Sample 3b**. The Institutional Review Board of the Boston University Medical Center approved the study protocol and all study participants provided written informed consent.

### Cardiovascular Health (CVH) Score

A CVH Score for each participant was derived using the seven CVH metrics (see S1 Table). As previously described [2, 12] each CVH metric is assigned 0, 1, or 2 points to characterize poor, intermediate, or ideal health categories, respectively, and all points are summed to designate an overall CVH score, ranging from 0 (indicating extremely poor CVH) to 14 points (indicating ideal CVH). Participants with CVH scores of 0 to 7, 8 to 11, or 12–14 points were categorized as having poor, intermediate, or ideal CVH, respectively [2, 12]. In the current investigation, participants with intermediate or ideal CVH (8–11 and 12–14 points, respectively) were compared to those who had “poor CVH” (0–7 points).

At each examination cycle, participants underwent measurements of resting systolic and diastolic BP, height, weight, total cholesterol level, and fasting serum glucose level, as previously described [30–32]. Smoking status, diet, and physical activity were self-reported. Participants designated their smoking status as current smoker, former, or never smoker. Diet quality was assessed through the use of a validated food frequency questionnaire (FFQ) administered at the time of exam which captured consumption of the following components: fruits and vegetables (≥4.5 cups/day), fish (≥2 3.5-oz servings/week), sodium (<1500 mg/day), sugar-sweetened beverages (≤450 kcal/week), and fiber-rich whole grains (≥3 1-oz equivalent servings/day) [1]. Diet quality was categorized as poor, intermediate, or ideal if participants met 0, 1, or ≥2 healthy components, respectively, of the FFQ consistent with prior reports [12, 14, 33]. Physical activity was self-reported via a validated questionnaire and the physical activity index was calculated as follows for FOS: 28*(Flight of stairs climbed each day) + 56*(Number of city blocks walked each day) + 4.5*(Number of times/week engaged in intense physical activity)*60, and as follows for Gen3: 1*(sleep hours/day) + 1.1*(sedentary hours/day) + 1.5*(slight activity hours/day + 2.4*(moderate activity hours/day) + 5*(heavy activity hours/day). Higher quartiles of the physical activity index indicate higher physical activity. Participants in the highest quartile were categorized in the ideal status of physical activity, those in the third quartile were categorized in the intermediate status, and those below the median were categorized in the poor status.

### Subclinical Disease (SubDz) components

Participants underwent testing for numerous SubDz components in the FOS and Gen3 cohorts, consistent with prior studies [22, 34]. Presence of SubDz was defined as having at least one of the following: left ventricular hypertrophy (LVH), increased carotid artery intimal-media thickness (CIMT), microalbuminuria (MA), reduced ankle brachial index (ABI), or presence of coronary artery calcification (CAC) (S2 Table). CIMT was not available in Gen3 participants.

Standard two-dimensional transthoracic echocardiography was performed on all participants. All echocardiograms were read in a blinded fashion by a cardiologist or sonographer experienced in echocardiography with excellent reproducibility of all measurements, as previously reported [35]. In accordance with American Society of Echocardiography (ASE) guidelines, the thickness of the left ventricular (LV) posterior wall at end-diastole as well as the interventricular septum were estimated from the average of ≥3 cardiac cycles measured with a digital M-mode [36]. LVH was defined using ASE criteria as having LV mass indexed to body surface area >95 g/m^2^ for women and >115 g/m^2^ for men. Carotid ultrasonography was performed using a standard protocol [22, 34]. Measurements of CIMT were made from gated diastolic images of the left and right carotid arteries at the level of the proximal 2-cm of the internal carotid artery, the distal common carotid artery, and the carotid artery bulb. Increased CIMT was defined as having a standardized CIMT ≥80^th^ sex-specific percentile in the sample or extreme increase of the common carotid artery intimal-medial thickness ≥1 mm [22, 34]. Ankle-brachial systolic BP values were obtained through use of an 8-MHz Doppler pen probe and ultrasonic Doppler flow detector (Parks Medical Electronics, Aloha, OR) using previously described standardized protocols [26]. MA was measured with an immunoturbidimetric assay (Tina-Quant Albumin Assay; Roche Diagnostics) and indexed to urinary creatinine, using the modified Jaffe method, to estimate the urine albumin-to-creatinine ratio (UACR) in a spot urine sample. Presence of MA was defined as UACR ≥25 mg/g for women and UACR ≥30 mg/g for men. Coronary artery calcium was measured according to previously described study protocols [37]. Non-contrast scans utilized an 8-slice Multi-Detector Computed Tomography (MDCT) scanner (LightSpeed Ultra; General Electric, Milwaukee, WI). Images were acquired with prospective electrocardiographic triggering during a single mid-inspiratory breath hold. For each study, a modified Agatston Score (AS) was generated through summation of calcified lesions, defined as an area of ≥3 connected pixels with a CT attenuation of >130 Hounsfield units, in each individual cross section. An AS ≥100 Agatston units was used to define the presence of CAC.

### Cardiovascular disease (CVD)

Clinical CVD was defined as a composite of coronary heart disease (myocardial infarction, coronary insufficiency, and angina pectoris), stroke or transient ischemic attack, intermittent claudication, or heart failure consistent with prior Framingham publications [38]. A panel of three physicians adjudicated all CVD events using standardized definitions for these events. Incident CVD events were captured through December 31, 2016.

### Statistical analysis

We used binary measures to define the presence or absence of each SubDz component (LVH, CIMT, ABI, MA, and CAC), incident CVD, and ideal, intermediate or poor CVH pooled between FOS and Gen3 participants. We generated cross-tabulated frequencies and percentages for each of the three paradoxes (CVH-SubDz, CVH-CVD, SubDz-CVD). For evaluating the CVH-CVD and SubDz-CVD paradoxes, we also generated pooled frequencies and percentages of ideal/intermediate/poor CVH and each SubDz component stratified by incident CVD. For the CVH-SubDz and SubDz-CVD paradoxes, we evaluated each SubDz component separately. For the CVH-CVD and SubDz-CVD paradoxes, we generated incidence rates for CVD stratified by CVH and SubDz status, separately. Additional sensitivity analyses stratifying the presence of these paradoxical relations by sex and median age were performed. Lastly, we used logistic regression models to estimate age- and sex-adjusted mean predicted probabilities of incident CVD, stratified by CVH status and by SubDz component. All analyses were conducted using SAS 9.4 (SAS Institute Inc., Cary, NC).

## Results

Baseline characteristics of the study sample by cohort are summarized in Table 1.

**Table 1:** Characteristics of study sample.

Characteristics	Offspring (n = 3,532)	Third Generation (n = 4,095)
Age, years	59 ± 10	40 ± 9
Women, n (%)	1875 (53)	2183 (53)
Body mass index, kg/m^2^	27.9 ± 5.1	26.9 ± 5.6
Systolic blood pressure, mm Hg	128 ± 19	117 ± 14
Diastolic blood pressure, mm Hg	75 ± 10	75 ± 10
Hypertension, n (%)	1466 (42)	679 (17)
Hypertension treatment, n (%)	998 (28)	345 (9)
Total/HDL cholesterol ratio	4.4 ± 1.8	3.8 ± 1.4
Total cholesterol, mg/dL	106 ± 40	189 ± 36
Lipid-lowering treatment, n (%)	456 (13)	273 (7)
Serum creatinine, mg/dL	1.16 ± 0.24	0.80 ± 0.16
Current smoking, n (%)	540 (15)	635 (16)
Diabetes mellitus, n (%)	357 (10)	123 (3)
Diabetes treatment, n (%)	198 (6)	78 (2)
CVH score, n (%)^a^		
Poor (0–7)	556 (25)	330 (10)
Intermediate (8–11)	1362 (60)	1695 (51)
Ideal (12–14)	350 (15)	1303 (39)
Diet score	1.56 ± 0.91	1.52 ± 0.97
Coronary artery calcium score, Agatston units	36 (0, 248)	0 (0, 1.4)
Coronary artery calcium score (≥100 AU), n (%)	499 (38)	156 (8)
Ankle brachial index ratio	1.13 (1.06, 1.19)	1.19 (1.13, 1.24)
Ankle brachial index ratio (< 0.9), n (%)	129 (4.0)	35 (1.3)
Urine albumin/creatinine ratio, mg/g	6.4 (2.8, 15.4)	4.0 (2.6, 7.6)
Microalbuminuria, n (%)	349 (12)	112 (3)
Left ventricular mass indexed by body surface area, g/m^2^	84 (74, 97)	81 (71, 93)
Left ventricular hypertrophy, n (%)^**b**^	428 (16)	225 (6)
Carotid IMT, mm^**c**^	0.56 (0.48, 0.72)	N/A
Increased carotid IMT, n (%)^**c**^	675 (20)	N/A
Prevalent CVD, n (%)	413 (12)	66 (2)

**Note:** Data are presented as mean ± standard deviation or median (Q1, Q3) unless otherwise noted.

**Abbreviations:** AHA, American Heart Association; AU, Agatston units; CVD, cardiovascular disease; CVH, cardiovascular health; HDL, high-density lipoprotein; LV, Left ventricular; IMT, intimal-medial thickness.

^a^Physical activity index defined by quartiles and diet score defined by number of healthy components (see S1 Table).

^**b**^LV hypertrophy defined as LV mass indexed to body surface area >95 g/m^2^ for women and >115 g/m^2^ for men.

^**c**^Carotid IMT data are not available in Third Generation cohort.

Most FOS participants had intermediate CVH (60%); 15% had ideal CVH. Slightly more than half of Gen3 participants had intermediate CVH (51%); 39% had ideal CVH.

### CVH–CVD paradox

**Fig 1:**
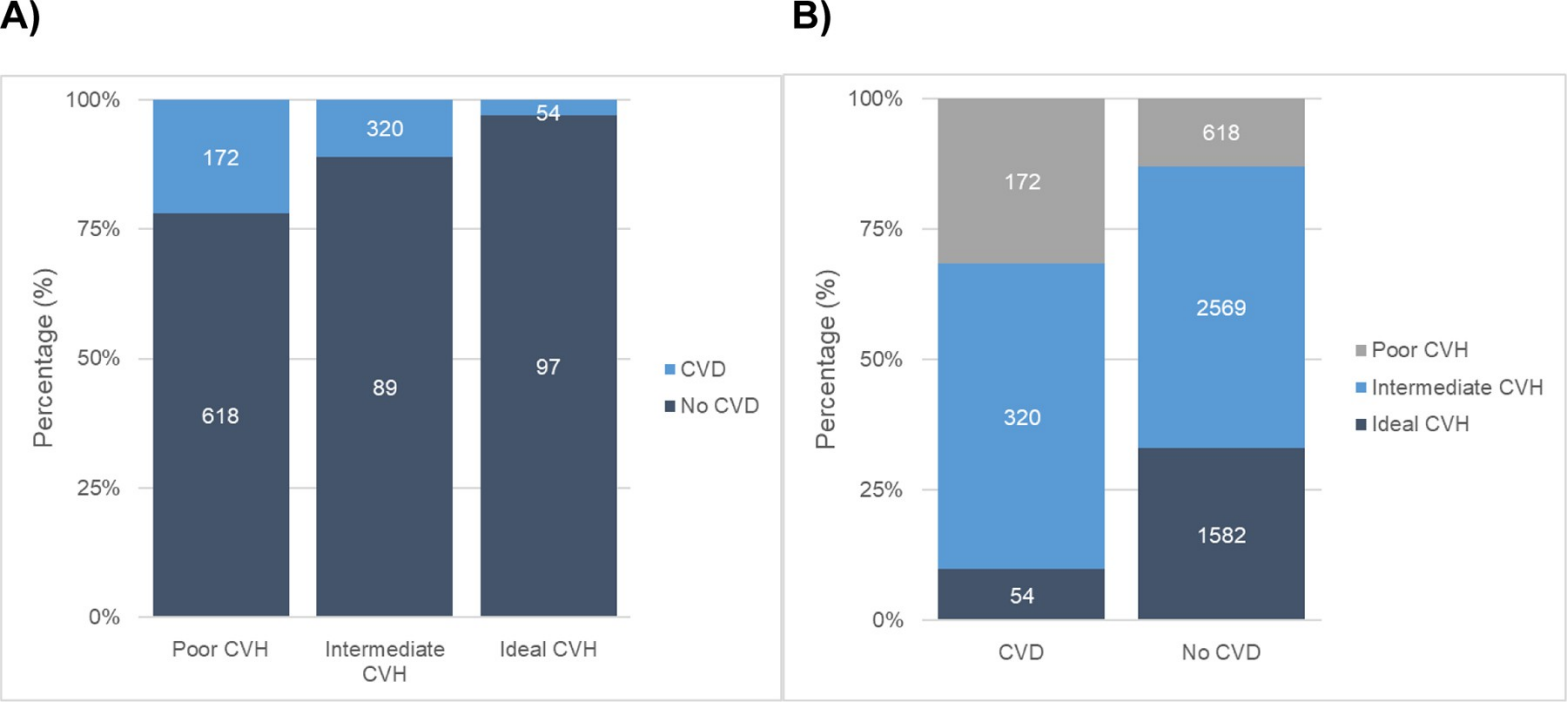
Pooled frequencies and percentages of: (A) Incident CVD by CVH status and (B) CVH status by incident CVD.

Using **Samples 1a and 1b** (n = 5,315), 85% of participants had intermediate or ideal CVH and we observed 546 (10%) incident CVD events (43% in women) over a median follow-up of 13.6 years (range: 0.001–22 years). A notable proportion of participants exhibited paradoxical phenomena between CVH status and incident CVD (Fig 1A and 1B), e.g., among participants with incident CVD, the majority (68%) had intermediate or ideal CVH. Among participants with poor CVH, most (78%) did not develop CVD.

We observed a higher incidence rate and mean age- and sex-adjusted predicted probability of CVD among those with poor CVH compared with those with intermediate or ideal CVH (Table 2). We also observed a lower odds of CVD with intermediate or ideal CVH compared with poor CVH (Table 2).

**Table 2:** Incidence rates and mean predicted probabilities of incident CVD stratified by CVH status and presence of each Subclinical Disease component.

		CVD N (%)	No CVD N (%)	Total	Crude Incidence Rate (95% CI)	Mean Predicted Probability of CVD	Odds Ratio (95% CI)
**CVH-CVD**							
**Poor CVH**		172 (31)	618 (13)	790	15.8 (13.6, 18.4)	21.8%	Reference
**Intermediate CVH**		320 (59)	2569 (54)	2889	7.7 (6.9. 8.6)	11.1%	0.62 (0.49, 0.77)
**Ideal CVH**		54 (10)	1582 (33)	1636	2.4 (1.8, 3.1)	3.3%	0.32 (0.23, 0.45)
**Total**		546	4769				
**SubDz-CVD**							
**CIMT**	No SubDz	448 (67)	2049 (88)	2497	11.0 (10.0, 12.1)	17.9%	Reference
	SubDz	223 (33)	279 (12)	502	35.6 (31.2, 40.5)	44.4%	2.46 (1.97, 3.06)
	Total	671	2328				
**LVH**	No SubDz	468 (79)	5213 (92)	5681	5.8 (5.3, 6.4)	8.2%	Reference
	SubDz	128 (21)	427 (8)	555	17.1 (14.4, 20.4)	23.1%	1.63 (1.28, 2.09)
	Total	596	5640				
**MA**	No SubDz	600 (86)	5702 (95)	6302	6.8 (6.3, 7.4)	9.5%	Reference
	SubDz	96 (14)	271 (5)	367	19.7 (16.2, 24.1)	26.2%	1.71 (1.29, 2.26)
	Total	696	5973				
**ABI**	No SubDz	591 (95)	4733 (99)	5324	10.6 (9.7, 11.4)	11.1%	Reference
	SubDz	28 (5)	60 (1)	88	37.2 (25.7, 53.9)	31.8%	1.87 (1.12, 3.11)
	Total	619	4793				
**CAC**	No SubDz	139 (46)	2575 (87)	2714	3.7 (3.2, 4.4)	5.0%	Reference
	SubDz	161 (54)	399 (13)	560	22.7 (19.5, 26.5)	28.7%	4.03 (2.96, 5.49)
	Total	300	2974				

**Note:** Incidence rates are reported per 1000 person-years. Predicted probability and odds ratio is adjusted for age and sex.

Data reflect the pooled sample including Offspring and Third Generation cohorts.

**Abbreviations:** ABI, ankle brachial index; CAC, coronary artery calcium; CIMT, carotid intimal medial thickness, CVD, cardiovascular disease; CVH, cardiovascular health; LVH, left ventricular hypertrophy; MA, microalbuminuria; SubDz, subclinical disease.

### CVH–SubDz paradox

**Fig 2:**
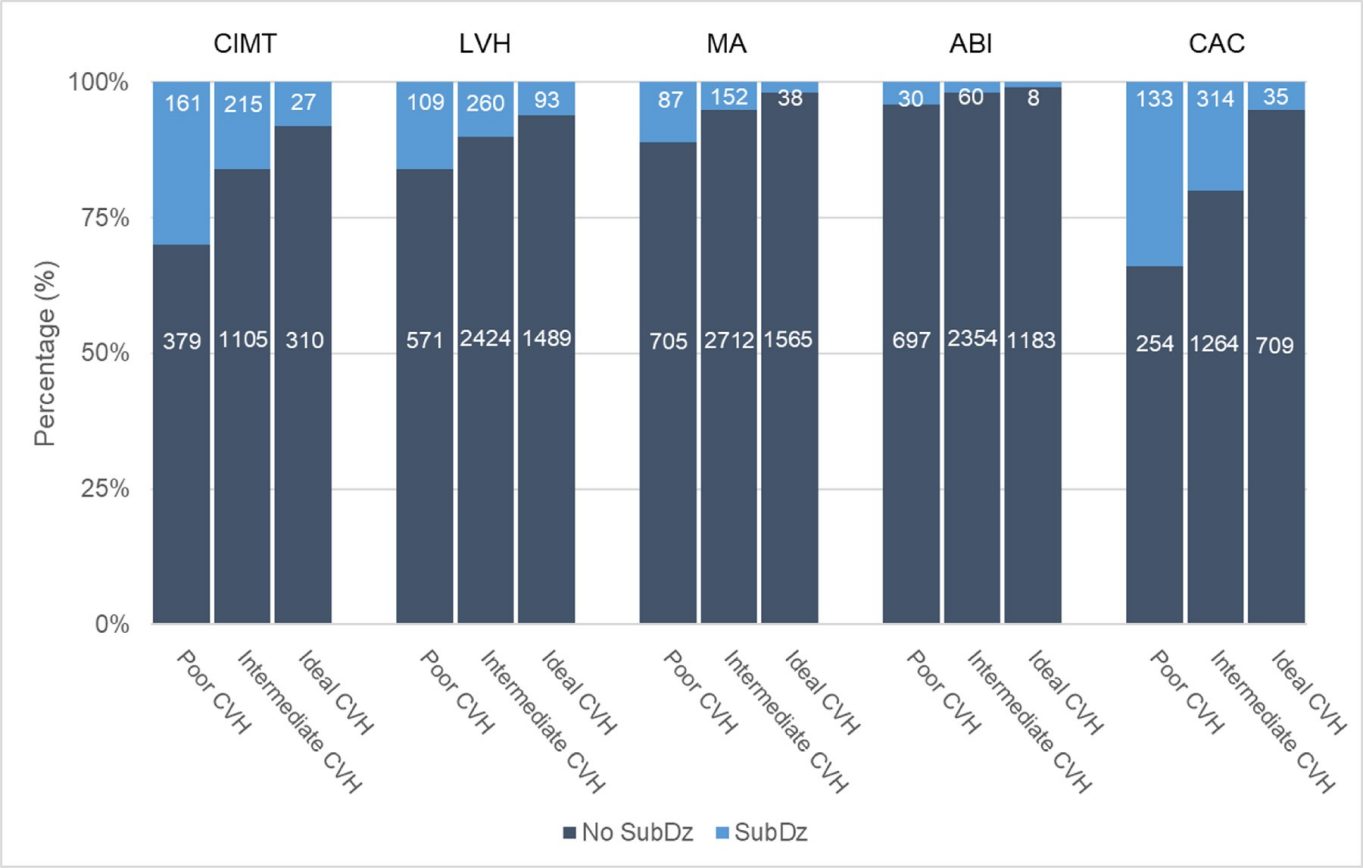
Pooled frequencies and percentages of present SubDz by CVH status (FOS and Gen3).

Using **Samples 2a and 2b** (n = 5,777), we observed that among those with poor CVH, the majority did not have concurrent abnormal CIMT, LVH, MA, ABI, or CAC (Fig 2). The absence of SubDz among those with poor CVH ranged from 66% for CAC to 96% for ABI (S3 Table).

### SubDz–CVD paradox

**Fig 3:**
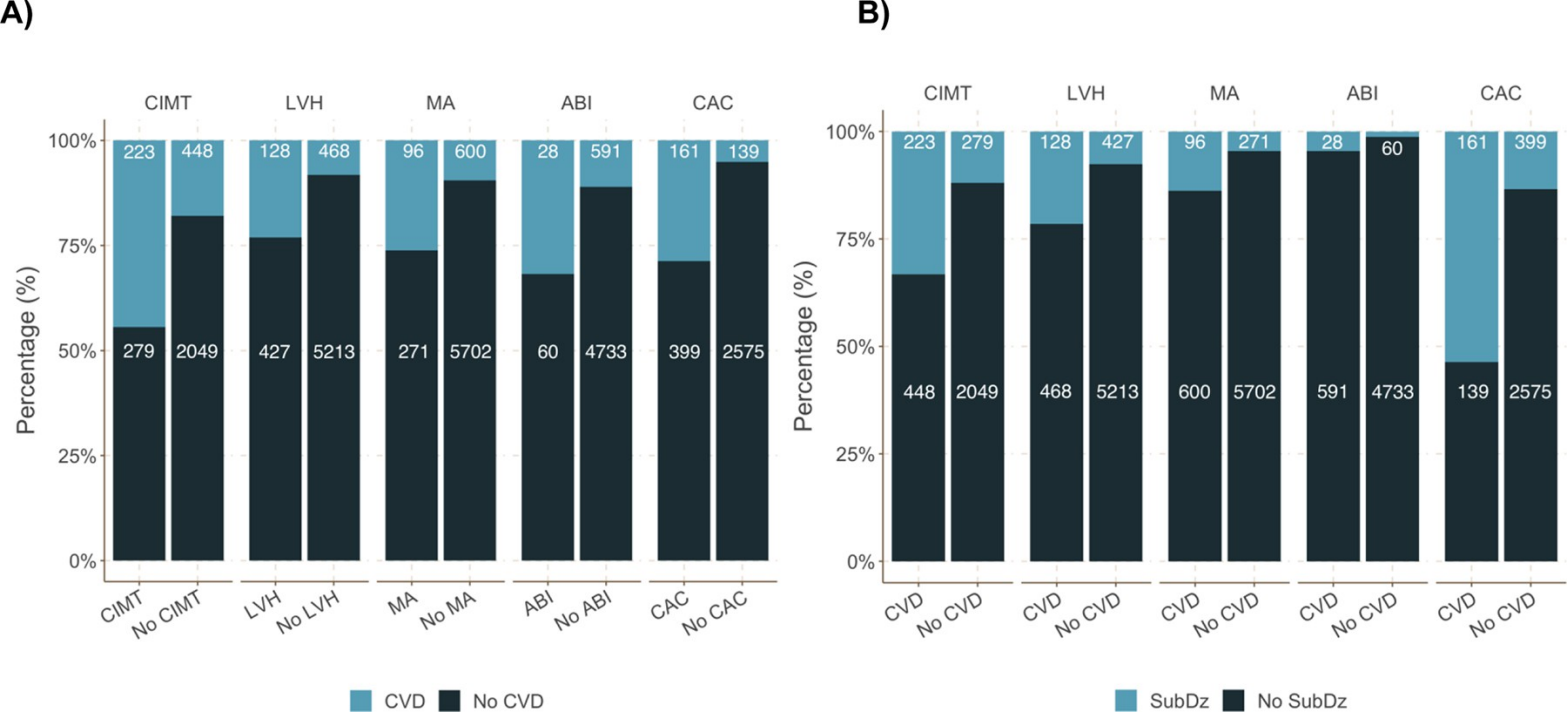
Pooled frequencies and percentages of: (A) Incident CVD by SubDz and (B) present SubDz by incident CVD.

Using **Samples 3a and 3b** (n = 7,297), 1,663 (23%) participants had prevalent SubDz and we observed 842 (12%) incident CVD events. The prevalence of SubDz ranged from 2% for ABI to 17% for CIMT and CAC (S4 Table). Among participants with prevalent SubDz, the majority did not develop CVD (Fig 3A). Among individuals with incident CVD, the majority did not have prevalent SubDz (Fig 3B). Notably, CAC was present in 54% of participants with incident CVD. Furthermore, among people with SubDz, most did not develop incident CVD on follow-up (ranging from 56% to 87%). Across all SubDz components, we observed higher incidence rates, mean predicted probabilities and odds of CVD among participants with prevalent SubDz compared with participants without SubDz (Table 2).

We also observed that the aforementioned three paradoxical relations were present in men and women separately (S3A Fig), as well as in younger and older age groups (defined using the median age of 46 in our sample; S3B Fig). Notably, the CVH-CVD paradox was more prevalent in younger individuals.

## Discussion

### Principal findings

First, most participants in the current investigation had intermediate or ideal CVH and were free of SubDz. Second, participants with poor CVH or prevalent SubDz had higher CVD incidence rates and mean predicted probabilities for CVD compared to participants with intermediate or ideal CVH or absent SubDz. Third, we observed the presence of CVH-CVD, SubDz-CVD, and CVH-SubDz paradoxes in a notable proportion of individuals, regardless of sex or age group. These paradoxical relations are underscored by the finding that the majority of individuals with incident CVD either had intermediate or ideal CVH or lack of SubDz.

### Comparison with the published literature

The majority of participants in our investigation had intermediate or ideal CVH, yet the prevalence of ideal CVH (≥12 points) using our composite 14-point definition, was low among Offspring participants (15%). Our definition of intermediate or ideal CVH (8–14), concordant with prior reports [2, 12], includes many individuals with non-ideal health metrics; this definition deviates from the one proposed by the AHA and may partially account for the observed paradoxes in SubDz and CVD associated with intermediate or ideal CVH in our sample. Moreover, the challenges of meeting the AHA strategic definition worldwide have been underscored by the low prevalence of ideal CVH, defined by meeting 6–7 ideal CVH metrics, in both US (ranging from 0.5% to 12%) and non-US (ranging from 0.3% to 12%) populations [10].

In recent clinical guidelines, CAC has emerged as an endorsed risk stratification tool [39]. Notably, in the present investigation, only 15% of participants with intermediate or ideal CVH had non-zero CAC scores, a marker of SubDz, underscoring the CVH-SubDz paradox. In a report using MESA data, more than 40% of adults with intermediate or ideal CVH had non-zero CAC scores [4] and nearly 80% of participants with poor CVH did not develop CVD (CVH-CVD paradox), similar to our findings. Our investigation included assessment of a comprehensive panel of SubDz components; it is noteworthy that the presence of the CVH-SubDz paradox was consistent across all SubDz components. Furthermore, a prior FHS report noted that adjusting for prevalent SubDz and serum biomarkers only slightly attenuated the inverse association of CVH score with CVD [5]. Taken together with the results from our investigation, these findings suggest that additional biologic mechanisms for developing CVD among individuals with intermediate or ideal CVH remain to be elucidated.

Moreover, in our investigation, the majority of incident CVD events occurred in individuals with intermediate or ideal CVH or absent SubDz, with the exception of CAC, highlighting the presence of this paradoxical phenomenon, whereby larger numbers of people with low or moderate risk produce a greater amount of CVD cases compared to smaller numbers of high-risk individuals [40]. Our results emphasize that neither intermediate or ideal CVH nor absent SubDz guarantees protection from CVD development, and vice-versa. Nevertheless, achieving ideal CVH and risk factor control among the general population remains a noteworthy goal. Improved CVD outcomes have been reported with increasing antecedent time spent in intermediate or ideal CVH in adulthood [12]. The low prevalence of ideal CVH in the community poses a challenge in evaluating for incremental benefits of achieving ideal CVH over intermediate CVH [2, 4, 12, 41]. Additional studies are warranted to elucidate the mechanisms underlying the paradoxical phenomena we highlighted in our investigation.

### Strengths and limitations

This investigation was performed in a large community-based sample of middle-aged adults who were under continuous surveillance for the development of CVD events. The longitudinal follow-up of our study sample facilitated a precise determination of CVH scores and adjudication of CVD outcomes. In addition, we evaluated a comprehensive set of SubDz components. Several limitations to this study warrant consideration. First, CVH scores were assessed at a single examination; as such, duration of time spent in ideal, intermediate, or poor CVH or changes in CVH scores over time were not assessed. Second, we used definitions of physical activity and diet quality in our study to maintain consistency with prior FHS publications, yet these definitions were different from those advocated in the AHA Life’s Simple 7. Lastly, our sample includes predominantly Caucasian, European-ancestry, middle-aged adults, limiting generalizability of our results to other multi-ethnic cohorts or age groups.

## Conclusions

Our findings indicate that the paradoxical phenomena involving CVH, SubDz, and, CVD are frequent in the community. The majority of incident CVD occurred in participants with intermediate or ideal CVH or in those who lacked evidence of SubDz. These findings highlight a need for further studies to elucidate potential mechanisms involved in these paradoxical phenomena and better ways to identify CVD susceptibility in people with intermediate or ideal CVH.

## Supporting information

S1 Table Definition of cardiovascular health (CVH) score. (DOCX)

S2 Table Definition of subclinical disease components. (DOCX)

S3 Table Pooled frequencies and percentages of CVH status with present SubDz (FOS and Gen3). (DOCX)

S4 Table Pooled frequencies and percentages of incident CVD with present SubDz (FOS and Gen3). (DOCX)

S1 Fig Flow diagram of study sample in the Framingham Offspring cohort. (DOCX)

S2 Fig Flow diagram of study sample in the Framingham Third Generation cohort. (DOCX)

S3 Fig CVH-SubDz-CVD Paradox by A) sex and B) age groups (median cut-off).
(DOCX)
