# Supplementary material for: Notable paradoxical phenomena in associations between cardiovascular health score, subclinical and clinical cardiovascular disease in the community: The Framingham Heart Study
Source: PLoS One. 2022 May 5;17(5):e0267267. doi: 10.1371/journal.pone.0267267 (PMC9070900; doi:10.1371/journal.pone.0267267)
Supplement: S1 Table — (DOCX) [file pone.0267267.s001.docx]

**S1 Table. Definition of Cardiovascular Health (CVH) Score**

| **CVH Score Component** | **Poor Health**  **(0 points)** | **Intermediate Health**  **(1 point)** | **Ideal Health**  **(2 points)** |
| --- | --- | --- | --- |
| Smoking | Current smoker | Former smoker (quit >12 months ago) | Never smoker |
| Physical Activity^a^ | Below median | Third quartile | Top quartile |
| Diet Score^b^ | 0 component | 1 component | ≥2 components |
| Body Mass Index | ≥30 kg/m^2^ | 25-30 kg/m^2^ | <25 kg/m^2^ |
| Blood Pressure | SBP≥140 or DBP≥90 mm Hg | SBP 120-140 mm Hg or DBP 80-90 mm Hg or on antihypertensive medication | SBP/DBP <120/80 mm Hg & no antihypertensive medication |
| Fasting Plasma Glucose | ≥126 mg/dL | 100-126 mg/dL or on treatment for diabetes to goal | <100 mg/dL & no treatment for diabetes |
| Total Serum Cholesterol | ≥240 mg/dL | 200-240 mg/dL or on dyslipidemia treatment | <200 mg/dL & no treatment for dyslipidemia |

^a^Physical activity score was calculated using the formula:
For Third Generation: 1*sleep hours/day + 1.1*sedentary hours/day + 1.5*slight activity hours/day + 2.4*moderate activity hours/day + 5*heavy activity hours/day.
For Offspring cohort: 28*Flight of stairs climbed each day + 56*Number of city blocks walked each day + 4.5*Number of times/week engaged in intense physical activity*60. The top quartile of this score was used to indicate ideal physical activity, which corresponds qualitatively to the definition used by the American Heart Association.

^b^Healthy diet score components: ≥4.5 cups/day fruits and vegetables, ≥2 3.5-oz servings/week fish, <1500 mg/day sodium, ≤450 kcal/week sugar-sweetened beverages, and ≥3 1-oz equivalent servings/day fiber-rich whole grains.
