# Supplementary material for: Notable paradoxical phenomena in associations between cardiovascular health score, subclinical and clinical cardiovascular disease in the community: The Framingham Heart Study
Source: PLoS One. 2022 May 5;17(5):e0267267. doi: 10.1371/journal.pone.0267267 (PMC9070900; doi:10.1371/journal.pone.0267267)
Supplement: S2 Table — (DOCX) [file pone.0267267.s002.docx]

**S2 Table. Definition of subclinical disease components**

| Component | Definition | Cut-points for subclinical disease used in present study |
| --- | --- | --- |
| **1. LV hypertrophy** | | |
| LV hypertrophy by echocardiography | LV mass was calculated as 0.8 { 1.04 [ (IVS+LVEDD+PW)^3 - (LVEDD)^3 ] } + 0.6 g. LV mass values were then indexed to body surface area. Body surface area was calculated as 0.007184*(wgt_kg^(0.425))* (hgt_cm^(0.725)). | LVM indexed to body surface area >95 g/m^2^ for women and >115 g/m^2^ for men |
| **2. Carotid ultrasound abnormality** | | |
| Increased carotid artery IMT | A composite measure that combined the maximal common carotid artery IMT and maximal internal carotid artery IMT was obtained by averaging these two measurements after standardization (subtraction of the mean and division by the standard deviation for the measurement). | 1) A standardized carotid IMT that met or exceeded the sex-specific 80th percentiles in the sample; or  2) An extreme increase of common carotid IMT |
| Extreme increase in common carotid artery IMT | An extreme increase of common carotid IMT ≥1 mm. |  |
| **3. Peripheral arterial disease** | | |
| Ankle-brachial index ≤0.9 | Defined as the ratio of the average systolic blood pressure at the ankle of each leg divided by the average systolic blood pressure in the arm with the highest blood pressure. | An ankle-brachial index at or below 0.9 in either leg. |
| **4. Glomerular endothelial dysfunction** | | |
| Microalbuminuria | Urine albumin to urine creatinine ratio | ≥25 μg/mg in men, and ≥35 μg/mg in women |
| **5.Coronary artery calcium** | | |
| Coronary artery calcium | Agatston score (AS) calculated by multiplying the area of each calcified lesion with a weighted attenuation score dependent on the maximal attenuation. | AS ≥100 |

**Abbreviations:** IMT, intimal medial thickness; IVS, interventricular septum; LV, left ventricular; LVEDD, left ventricular end-diastolic dimension; LVM, left ventricular mass; PW, posterior wall.
