# Supplementary material for: Notable paradoxical phenomena in associations between cardiovascular health score, subclinical and clinical cardiovascular disease in the community: The Framingham Heart Study
Source: PLoS One. 2022 May 5;17(5):e0267267. doi: 10.1371/journal.pone.0267267 (PMC9070900; doi:10.1371/journal.pone.0267267)
Supplement: S3 Table — (DOCX) [file pone.0267267.s003.docx]

**S3 Table. Pooled frequencies and percentages of CVH status with present SubDz (FOS and Gen3)**

| **SubDz** | | **Poor CVH** | **Row Percent** | **Good CVH** | **Row Percent** | **Row Total** |
| --- | --- | --- | --- | --- | --- | --- |
| **CIMT** | Normal | 379 | 21.1% | 1415 | 78.9% | 1794 |
| Column Percent | | 70.2% |  | 85.4% |  |  |
|  | Abnormal | 161 | 40.0% | 242 | 60.0% | 403 |
| Column Percent | | 29.8% |  | 14.6% |  |  |
| **Column Total** | | 540 |  | 1657 |  |  |
| **LVH** | Normal | 571 | 12.7% | 3913 | 87.3% | 4484 |
| Column Percent | | 84.0% |  | 91.7% |  |  |
|  | Abnormal | 109 | 23.6% | 353 | 76.4% | 462 |
| Column Percent | | 16.0% |  | 8.3% |  |  |
| **Column Total** | | 680 |  | 4266 |  |  |
| **MA** | Normal | 705 | 14.2% | 4277 | 85.8% | 4982 |
| Column Percent | | 89.0% |  | 95.7% |  |  |
|  | Abnormal | 87 | 31.4% | 190 | 68.6% | 277 |
| Column Percent | | 11.0% |  | 4.3% |  |  |
| **Column Total** | | 792 |  | 4467 |  |  |
| **ABI** | Normal | 697 | 16.5% | 3537 | 83.5% | 4234 |
| Column Percent | | 95.9% |  | 98.1% |  |  |
|  | Abnormal | 30 | 30.6% | 68 | 69.4% | 98 |
| Column Percent | | 4.1% |  | 1.9% |  |  |
| **Column Total** | | 727 |  | 3605 |  |  |
| **CAC** | Normal | 254 | 11.4% | 1973 | 88.6% | 2227 |
| Column Percent | | 65.6% |  | 85.0% |  |  |
|  | Abnormal | 133 | 27.6% | 349 | 72.4% | 482 |
| Column Percent | | 34.4% |  | 15.0% |  |  |
| **Column Total** | | 387 |  | 2322 |  |  |

Data reflect the pooled sample including Offspring and Third Generation cohorts.

**Abbreviations:** ABI, ankle brachial index; CAC, coronary artery calcium; CIMT, carotid intimal medial thickness; CVH, cardiovascular health; LVH, left ventricular hypertrophy; MA, microalbuminuria; SubDz; subclinical disease.
