# Supplementary material for: Notable paradoxical phenomena in associations between cardiovascular health score, subclinical and clinical cardiovascular disease in the community: The Framingham Heart Study
Source: PLoS One. 2022 May 5;17(5):e0267267. doi: 10.1371/journal.pone.0267267 (PMC9070900; doi:10.1371/journal.pone.0267267)
Supplement: S4 Table — (DOCX) [file pone.0267267.s004.docx]

**S4 Table. Pooled frequencies and percentages of incident CVD with present SubDz (FOS and Gen3)**

| **SubDz** | | **CVD** | **Row Percent** | **No CVD** | **Row Percent** | **Row Total** |
| --- | --- | --- | --- | --- | --- | --- |
| **CIMT** | Normal | 448 | 17.9% | 2049 | 82.1% | 2497 |
| Column Percent | | 66.8% |  | 88.0% |  |  |
|  | Abnormal | 223 | 44.4% | 279 | 55.6% | 502 |
| Column Percent | | 33.2% |  | 12.0% |  |  |
| **Column Total** | | 671 |  | 2328 |  |  |
| **LVH** | Normal | 468 | 8.2% | 5213 | 91.8% | 5681 |
| Column Percent | | 78.5% |  | 92.4% |  |  |
|  | Abnormal | 128 | 23.1% | 427 | 76.9% | 555 |
| Column Percent | | 21.5% |  | 7.6% |  |  |
| **Column Total** | | 596 |  | 5640 |  |  |
| **MA** | Normal | 600 | 9.5% | 5702 | 90.5% | 6302 |
| Column Percent | | 86.2% |  | 95.5% |  |  |
|  | Abnormal | 96 | 26.2% | 271 | 73.8% | 367 |
| Column Percent | | 13.8% |  | 4.5% |  |  |
| **Column Total** | | 696 |  | 5973 |  |  |
| **ABI** | Normal | 591 | 11.1% | 4733 | 88.9% | 5324 |
| Column Percent | | 95.5% |  | 98.7% |  |  |
|  | Abnormal | 28 | 31.8% | 60 | 68.2% | 88 |
| Column Percent | | 4.5% |  | 1.3% |  |  |
| **Column Total** | | 619 |  | 4793 |  |  |
| **CAC** | Normal | 139 | 5.1% | 2575 | 94.9% | 2714 |
| Column Percent | | 46.3% |  | 86.6% |  |  |
|  | Abnormal | 161 | 28.8% | 399 | 71.2% | 560 |
| Column Percent | | 53.7% |  | 13.4% |  |  |
| **Column Total** | | 300 |  | 2974 |  |  |

Data reflect the pooled sample including Offspring and Third Generation cohorts.

**Abbreviations:** ABI, ankle brachial index; CAC, coronary artery calcium; CIMT, carotid intimal medial thickness, CVD, cardiovascular disease; LVH, left ventricular hypertrophy; MA, microalbuminuria; SubDz; subclinical disease.
