# Supplementary material for: Notable paradoxical phenomena in associations between cardiovascular health score, subclinical and clinical cardiovascular disease in the community: The Framingham Heart Study
Source: PLoS One. 2022 May 5;17(5):e0267267. doi: 10.1371/journal.pone.0267267 (PMC9070900; doi:10.1371/journal.pone.0267267)
Supplement: S2 Fig — (DOCX) [file pone.0267267.s006.docx]

**S2 Fig. Flow diagram of study sample in the Framingham Third Generation cohort**


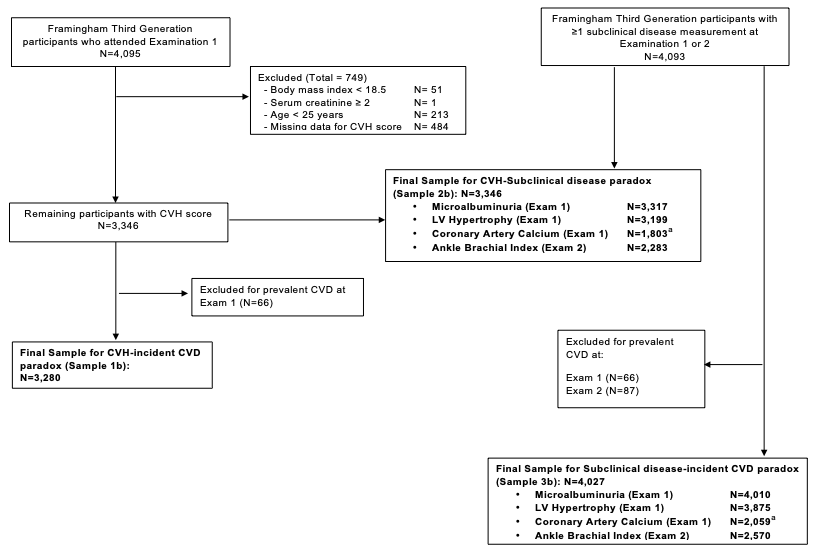


**Abbreviations:** CVD, cardiovascular disease; CVH, cardiovascular health; LV, left ventricular

**^a^**Coronary artery calcium measurements were only available on a limited number of participants
