# Supplementary material for: Notable paradoxical phenomena in associations between cardiovascular health score, subclinical and clinical cardiovascular disease in the community: The Framingham Heart Study
Source: PLoS One. 2022 May 5;17(5):e0267267. doi: 10.1371/journal.pone.0267267 (PMC9070900; doi:10.1371/journal.pone.0267267)
Supplement: S3 Fig — CVH-SubDz-CVD Paradox by A) sex and B) age groups (median cut-off). (DOCX) [file pone.0267267.s007.docx]

| **S3 Fig. CVH-SubDz-CVD Paradox by ) sex and B) age groups (median cut-off)**   1. **B)**   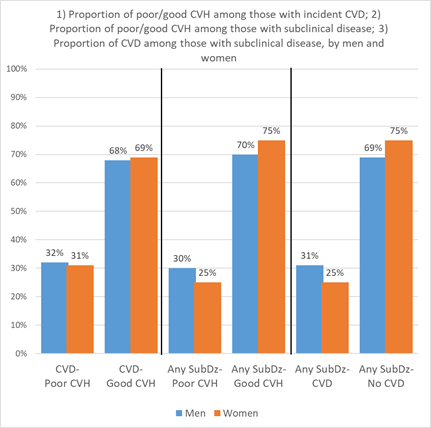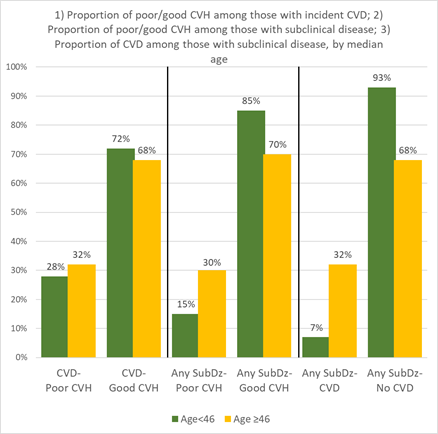 |
| --- |
|  |

**Abbreviations:** CVD, cardiovascular disease; LVH, left ventricular hypertrophy; MA, microalbuminuria; SubDz, subclinical disease.
